# Supplementary material for: Selective Heterogeneous Fenton Degradation of Formaldehyde Using the Fe-ZSM-5 Catalyst
Source: Molecules. 2024 Jun 19;29(12):2911. doi: 10.3390/molecules29122911 (PMC11206745; doi:10.3390/molecules29122911)
Supplement: Supplementary file 1 [file molecules-29-02911-s001.zip › molecules-2985643-Supplementary Materials.pdf]

In formaldehyde: glucose concentrations 20 mg L^{-1}: 100 mg L^{-1} (1/5), 60 mg L^{-1}: 100 mg L^{-1}(3/5), 100 mg L^{-1}: 100 mg L^{-1}(1/1), 150 mg L^{-1}: 100 mg L^{-1}(3/2), 200 mg L^{-1}: 100 mg L^{-1}(2/1) and the catalyst dose of 10 g L^{-1}, samples were taken every 60 min to determine the concentration. The effect of ZSM-5 molecular sieve on the adsorption capacity of formaldehyde and glucose was investigated under the condition of different proportions of formaldehyde and glucose mixed solution.

From Figure 1 to Figure 4, Table 1 and Table 2, the effects of ZSM-5 molecular sieve on glucose and formaldehyde in the two components can be seen. The adsorption was more consistent with the quasi-second-order kinetic equation.

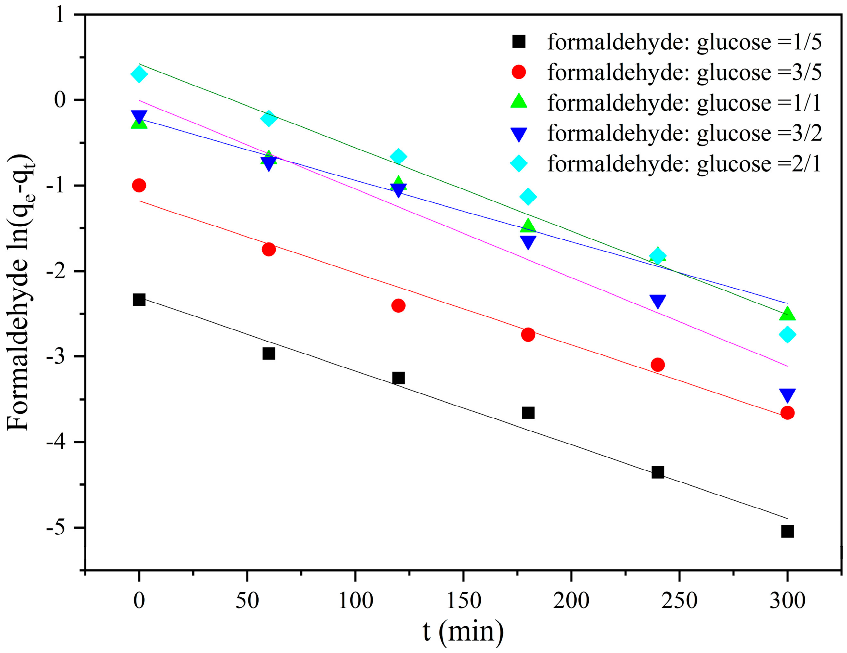

Figure S1: Quasi-first order kinetic adsorption equation fitting of formaldehyde in two components

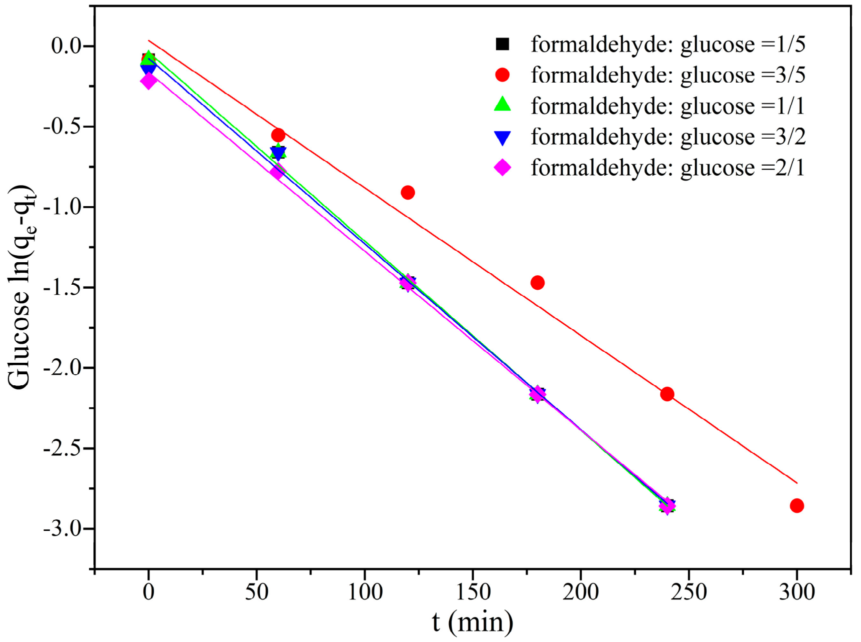

Figure S2: Quasi-first order kinetic adsorption equation fitting of glucose in two components

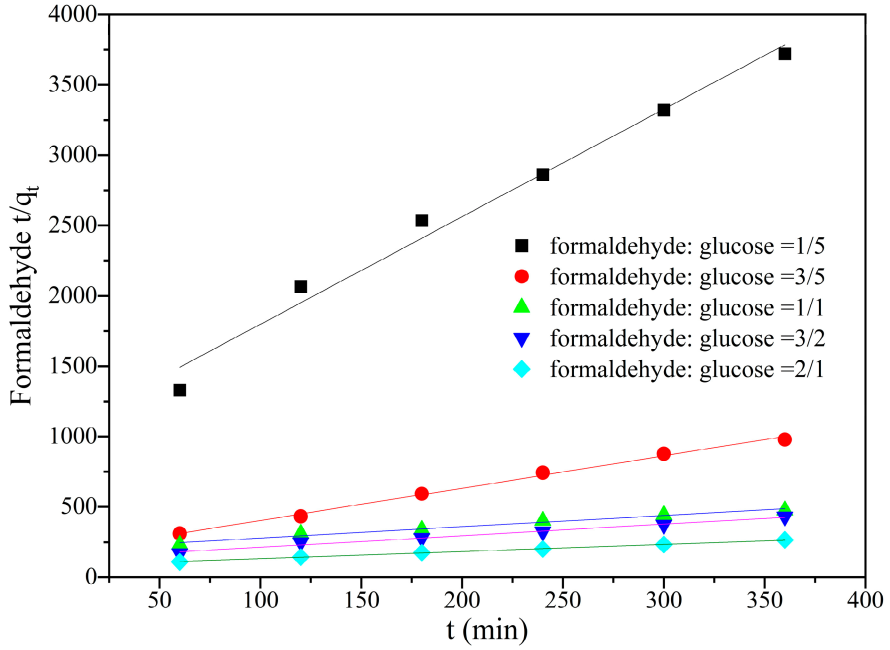

Figure S3: Quasi-second order kinetic adsorption equation fitting of formaldehyde in two components

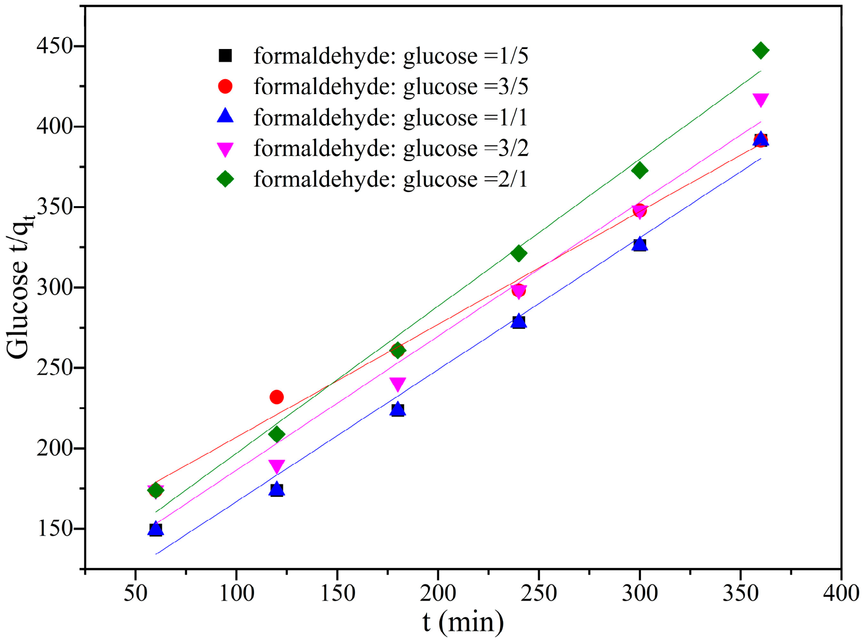

Figure S4: Quasi-second order kinetic adsorption equation fitting of glucose in two components

Table S1: The related parameters of glucose in two components were fitted by kinetic model

	Ratio of formaldehyde to glucose concentration	Quasi-first order adsorption kinetics			Quasi-second-order adsorption kinetics		
		$K_1(min^{-1})$	$q_e(mg/g)$	R^2	$K_2(min·g/mg)$	$q_e(mg/g)$	R^2
1	1/5	0.0118	0.9639	0.9974	0.0079	1.2187	0.9839
2	3/5	0.0092	1.0360	0.9813	0.0036	1.4259	0.9919
3	1/1	0.0118	0.9639	0.9974	0.0079	1.2187	0.9839
4	3/2	0.0115	0.9273	0.9952	0.0067	1.2001	0.9715
5	2/1	0.0111	0.8487	0.9979	0.0079	1.0940	0.9874

Table S2: The related parameters of formaldehyde in two components were fitted by kinetic model

	Ratio of formaldehyde to glucose concentration	Quasi-first order adsorption kinetics			Quasi-second-order adsorption kinetics		
		$K_1(min^{-1})$	$q_e(mg/g)$	R^2	$K_2(min·g/mg)$	$q_e(mg/g)$	R^2
1	1/5	0.0086	0.8487	0.9759	0.0564	0.1309	0.9797
2	3/5	0.0084	0.3075	0.9734	0.0305	0.4348	0.9949
3	1/1	0.0072	0.8021	0.9817	0.0032	1.2541	0.9772
4	3/2	0.0104	0.9936	0.9530	0.0051	1.2229	0.9847
5	2/1	0.0098	1.5232	0.9736	0.0033	1.9508	0.9988
